# Clinical Implications of Polypharmacy for Patients with New-Onset Atrial Fibrillation Based on Real-World Data: Observations from the Korea National Health Insurance Service Data

**DOI:** 10.31083/j.rcm2505164

**Published:** 2024-05-11

**Authors:** Hong-Ju Kim, Pil-Sung Yang, Daehoon Kim, Jung-Hoon Sung, Eunsun Jang, Hee Tae Yu, Tae-Hoon Kim, Hui-Nam Pak, Moon-Hyoung Lee, Boyoung Joung

**Affiliations:** ^1^Division of Cardiology, Department of Internal Medicine, Severance Cardiovascular Hospital, Yonsei University College of Medicine, 03722 Seoul, Republic of Korea; ^2^Department of Cardiology, CHA Bundang Medical Center, CHA University, 13496 Seongnam, Republic of Korea

**Keywords:** arial fibrillation, real-world data, polypharmacy, all-cause mortality, major bleeding, stroke, heart failure admission

## Abstract

**Background:**

Polypharmacy is commonly observed in atrial fibrillation (AF) and is associated with poorer clinical outcomes. Our study aimed to elucidate the polypharmacy prevalence, its associated risk factors, and its relationship with adverse clinical outcomes using a ‘real-world’ database.

**Methods:**

This study included 451,368 subjects without prior history of AF (median age, 54 [interquartile range, 48.0–63.0] years; 207,748 [46.0%] female) from the Korea National Health Insurance Service-Health Screening (NHIS-HealS) database between 2002 and 2013. All concomitant medications prescribed were collected, and the intake of five or more concomitant drugs was defined as polypharmacy. During the follow-up, all-cause death, major bleeding events, transient ischemic attack (TIA) or ischemic stroke, and admission due to worsened heart failure were recorded.

**Results:**

Based on up to 7.7 (6.8–8.3) years of follow-up and 768,306 person-years, there were 12,241 cases of new-onset AF identified. Among patients with new-onset AF (40.0% females, median age 63.0 [54.0–70.0] years), the polypharmacy prevalence was 30.9% (3784). For newly diagnosed AF, factors, such as advanced age (with each increase of 10 years, odds ratios (OR) 1.32, 95% confidence interval (CI) 1.26–1.40), hypertension (OR 4.00, 95% CI 3.62–4.43), diabetes mellitus (OR 3.25, 95% CI 2.86–3.70), chronic obstructive pulmonary disease (COPD) (OR 3.00, 95% CI 2.51–3.57), TIA/ischemic stroke (OR 2.36, 95% CI 2.03–2.73), dementia history (OR 2.30, 95% CI 1.06–4.98), end-stage renal disease (ESRD) or chronic kidney disease (CKD) (OR 1.97, 95% CI 1.38–2.82), and heart failure (OR 1.95, 95% CI 1.69–2.26), were found to be independently correlated with the incidence of polypharmacy. Polypharmacy significantly increased the incidence and risk of major bleeding (adjusted hazard ratio (aHR) 1.26, 95% CI 1.12–1.41). The study observed a statistically significant increase in the incidence of all-cause mortality, however, the risk for all-cause mortality elevated but did not show significance (aHR 1.11, 95% CI 0.99–1.24). The risk of stroke and admission for heart failure did not change with polypharmacy.

**Conclusions:**

In our investigation using data from a nationwide database, polypharmacy was widespread in new-onset AF population and was related to major bleeding events. However, polypharmacy does not serve as an independent risk factor for adverse outcomes, with exception of major bleeding event. For AF patients, ensuring tailored medication for comorbidities as well as reducing polypharmacy are essential considerations.

## 1. Introduction 

Atrial fibrillation (AF) is known as the most common sustained arrhythmia observed in clinical situation. This medical condition is associated with a significant increase in stroke or heart failure related mortality and morbidity. Furthermore, AF is a major driving factor behind substantial healthcare expenditures, thereby placing a considerable burden on the healthcare system [1, 2, 3, 4, 5]. The worldwide AF epidemic is mainly attributed to an increasing aging population [6]. Patients with AF are often older and more affected by concomitant cardiovascular (CV) and other conditions that affect their clinical course, leading to an increased risk of CV and all-cause death [1, 2, 3, 4, 5].

AF is well known to be associated with high morbidity and mortality, which is mainly due to the increased risk of stroke or systemic thromboembolic events. However, AF is also associated with a high incidence of comorbidities, including diabetes mellitus, hypertension, coronary artery disease, chronic kidney disease, valvular heart disease, obesity, and heart failure. The potential development of these comorbidities is greater in patients with AF than in the general population [3, 4]. As a result, patients with AF are often required to be prescribed various classes of medications to manage comorbid conditions, potentially leading to polypharmacy.

The term ‘polypharmacy’ has several definitions, encompassing aspects, such as the simultaneous administration of multiple classes of medication and the use of medications in an inappropriate manner. However, in a conventional context, polypharmacy is mainly defined as the concurrent use of five or more classes of medication. As expected, instances of polypharmacy were found to be more prevalent among patients aged 65 years or older, and previous reports revealed that the prevalence of polypharmacy ranges from 40 to 95% in the AF population [7, 8].

In a meta-analysis published recently, polypharmacy is common in the AF population and is associated with increased risk of clinical outcomes such as all-cause and cardiac death, bleeding, hospitalization due to heart failure, poorer quality of life, and reduced physical activity [9, 10].

The purpose of our investigation was to (i) examine the polypharmacy prevalence; (ii) identify the risk factors for polypharmacy; and (iii) understand its relationship with adverse clinical outcomes, including all-cause death, major bleeding, ischemic stroke, and admission for heart failure, in patients with new-onset AF using ‘real-world’ data.

## 2. Materials and Methods

### 2.1 Data Extraction

Our investigation relied on the Korean National Health Insurance Service-Health Screening (NHIS-HealS) cohort. The characteristics of this cohort are discussed in previous studies [11, 12]. Established in 2002 and populated up to 2013, this cohort comprised 514,764 Koreans between 40 and 80 years. The dataset comprises information on lifestyle habits and behaviors gleaned from questionnaires, and significant findings from health examinations. The cohort consisted of a 10% random sample from health screening conducted between 2002 and 2003, focusing on individuals aged 40 to 80, due to a small fraction of those under 40 and a low response rate from those over 80. The NHIS-HealS database includes the following datasets: (1) diagnostic information, and admission and treatment data employing the International Classification of Disease-10 (ICD-10) codes, (2) sociodemographic data, and (3) National Health Screening data [11]. All insured adults undergo a general health screening test every two years. The National Health check-up includes blood tests, chest radiography, medical history questionnaires, and physical examinations. Information regarding death (cause of death and date) was linked using personal identification numbers from Statistics Korea [11, 12]. This study received approval from the Yonsei University Health System’s Institutional Review, exempted the requirement for informed consent. (4-2023-0453) and due to its retrospective design and use of NHIS-HealS cohort data.

### 2.2 Study Cohort

**Fig. 1.**
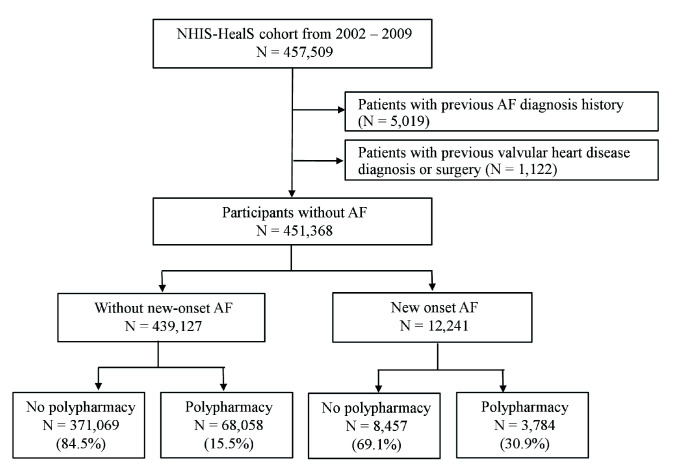
**Flow chart of the study population from the Korea National Health Insurance Service-Health Screening (NHIS-HealS) cohort**. Polypharmacy was defined as the concurrent use of five or more medications. AF, atrial fibrillation.

Initially, this study consisted of adults aged 40 to 80 years who underwent National Health checkups drawn from the NHIS-HealS cohort during the period 2002 to 2009 (n = 457,509) [13, 14]. We applied exclusion criteria as follows: (i) previous AF diagnosis history (n = 5109) and (ii) valvular heart disease, including mitral stenosis or prosthetic valve replacement status (ICD-10 codes: I050, I052, I342) (n = 1122). Finally, 451,368 participants without AF were included in the study (Fig. 1). Participants were followed-up until the end of 2015.

Comorbidity data in the NHIS database, which have been validated in previous studies, are provided in **Supplementary Table 1 **[3, 4, 5, 11, 13, 14, 15, 16, 17]. In the NHIS database, ICD-10 codes were used to define the comorbidities present at baseline. To guarantee data precision, new-onset AF was identified either by hospitalization or through a minimum of two distinct outpatient visits, as classified using ICD-10 code (I48), yielding a positive predictive value (PPV) reaching 94.1% [13]. The definition of polypharmacy was as the simultaneous use of five or more different medications [18].

### 2.3 Follow-Up and Clinical 

All-cause mortality was set as the primary outcome. The data of mortality was extracted from death registration which is administered by the National Population Registry of the Korea National Statistical Office [5, 11]. The secondary outcomes were ischemic stroke, major bleeding (defined as a composite of intracranial and gastrointestinal bleeding, with PPVs of 87.5% and 92.0%, respectively), and admission for heart failure. Information on the outcomes of interest in the NHIS data is provided in **Supplementary Table 2** and the validation method has been introduced in previous studies [5, 11].

### 2.4 Statistical Analysis

Continuous data were expressed as either mean ± standard deviation (SD) or median with interquartile ranges (IQRs), depending on normal distribution status, and categorical data as numbers and percentages. To compare these variables, we used student’s *t*-test and chi-square test for continuous and categorical data, respectively.

Logistic regression analysis was conducted to investigate the risk factors for polypharmacy; the results are expressed as odds ratios (OR) with 95% confidence intervals (CIs). Similarly, to establish the association between polypharmacy and adverse clinical outcomes, Cox proportional hazard regression analyses were performed. We conducted multivariable analysis with variables with *p*-value < 0.10 from the univariable analysis, and the results are expressed as the adjusted hazard ratio (aHR) with its 95% CI. The effect of new-onset AF was assessed using time-varying exposures.

The annual incidence rate with a 95% CI was assessed for patients with and without polypharmacy. This rate was calculated by dividing the number of adverse clinical outcomes by the total duration of patient exposure. Subsequently, the difference in the yearly event rates between the two groups and the statistical significance of this discrepancy were evaluated. Finally, the survival time distribution of the groups was compared using Kaplan-Meier analysis.

The *p*-values less than 0.05 indicated statistical significance, and we conducted analyses with performed R version 4.1.2 (R Foundation for Statistical Computing, Vienna, Austria; http://www.r-project.org/).

## 3. Results

### 3.1 Baseline Characteristics between No Polypharmacy and Polypharmacy

During the median follow up of 7.7 [6.8–8.3] years, 12,241 (2.7%) new-onset AF cases (median age of 63 [IQR 54.0–70.0]; 7342 [60.0%] men) occurred. The polypharmacy prevalence was 30.9% (n = 3784) among new-onset AF population and 15.5% (n = 68,058) among the no AF population (median age of 63 [IQR 54.0–70.0]; 7342 [60.0%] men). In the new-onset AF population, those with polypharmacy had a median of 7 (IQR 5–9) drugs, while those without polypharmacy used 0 (IQR 0–2) medications (*p* < 0.001). Patients with polypharmacy had a higher prevalence of several comorbidities, which was reflected via higher ${\mathrm{CHA}}_{2}$${\mathrm{DS}}_{2}$-VASc and HAS-BLED scores (Table 1).

**Table 1.** **Baseline characteristics between no polypharmacy and polypharmacy of the No AF and new-onset AF**.

		No AF			New-onset AF		
		No polypharmacy	Polypharmacy	*p*-value	No polypharmacy	Polypharmacy	*p*-value
		(N = 371,069)	(N = 68,058)		(N = 8457)	(N = 3784)	
Age, years		53.0 [48.0; 60.0]	62.0 [55.0; 69.0]	<0.001	60.0 [52.0; 68.0]	68.0 [60.0; 72.0]	<0.001
	Age ≥75 years	9502 (2.6)	5992 (8.8)	<0.001	696 (8.2)	588 (15.5)	<0.001
Female		167,418 (45.1)	35,431 (52.1)	<0.001	3163 (37.4)	1736 (45.9)	<0.001
Charlson comorbidity Index		1.0 [0.0; 2.0]	2.0 [1.0; 4.0]	<0.001	1.0 [0.0; 2.0]	3.0 [2.0; 5.0]	<0.001
${\mathrm{CHA}}_{2}$${\mathrm{DS}}_{2}$-VASc score		1.0 [0.0; 1.0]	2.0 [1.0; 4.0]	<0.001	1.0 [0.0; 2.0]	3.0 [2.0; 4.0]	<0.001
HAS-BLED score		0.0 [0.0; 1.0]	2.0 [1.0; 3.0]	<0.001	1.0 [0.0; 2.0]	2.0 [2.0; 3.0]	<0.001
BMI, kg/$m^{2}$		23.8 [21.9; 25.6]	24.4 [22.5; 26.5]	<0.001	24.0 [22.1; 26.0]	24.6 [22.4; 26.6]	<0.001
Systolic BP, mmHg		124.0 [113.0; 135.0]	130.0 [120.0; 140.0]	<0.001	130.0 [120.0; 140.0]	130.0 [120.0; 140.0]	<0.001
Diastolic BP, mmHg		80.0 [70.0; 85.0]	80.0 [70.0; 86.0]	<0.001	80.0 [70.0; 88.0]	80.0 [70.0; 89.0]	0.716
Smoking		68,881 (19.6)	9076 (14.1)	<0.001	1554 (19.5)	495 (13.9)	<0.001
Alcohol		99,716 (26.9)	12,346 (18.1)	<0.001	2528 (29.9)	693 (18.3)	<0.001
Medical history							
	Heart failure	5614 (1.5)	7049 (10.4)	<0.001	422 (5.0)	807 (21.3)	<0.001
	Hypertension	64,746 (17.4)	44,162 (64.9)	<0.001	2532 (29.9)	2879 (76.1)	<0.001
	Diabetes mellitus	17,556 (4.7)	18,204 (26.7)	<0.001	603 (7.1)	1020 (27.0)	<0.001
	Dyslipidemia	71,551 (19.3)	36,591 (53.8)	<0.001	2214 (26.2)	2114 (55.9)	<0.001
	Ischemic stroke or TIA	9189 (2.5)	11,125 (16.3)	<0.001	424 (5.0)	777 (20.5)	<0.001
	Previous MI	1640 (0.4)	2785 (4.1)	<0.001	116 (1.4)	269 (7.1)	<0.001
	Vascular disease	5891 (1.6)	6965 (10.2)	<0.001	304 (3.6)	522 (13.8)	<0.001
	Hyperthyroidism	7607 (2.1)	3355 (4.9)	<0.001	226 (2.7)	229 (6.1)	<0.001
	Hypothyroidism	7875 (2.1)	3408 (5.0)	<0.001	214 (2.5)	176 (4.7)	<0.001
	Osteoporosis	42,783 (11.5)	20,246 (29.7)	<0.001	1272 (15.0)	1144 (30.2)	<0.001
	Dementia history	380 (0.1)	532 (0.8)	<0.001	12 (0.1)	35 (0.9)	<0.001
	Hypertrophic cardiomyopathy	281 (0.1)	143 (0.2)	<0.001	61 (0.7)	38 (1.0)	0.132
	Pacemaker or ICD implantation	33 (0.0)	24 (0.0)	<0.001	10 (0.1)	1 (0.0)	0.215
	Anemia	35,968 (9.7)	10,271 (15.1)	<0.001	825 (9.8)	669 (17.7)	<0.001
	Hemorrhagic stroke history	980 (0.3)	868 (1.3)	<0.001	31 (0.4)	38 (1.0)	<0.001
	Major bleeding history	3306 (0.9)	1935 (2.8)	<0.001	122 (1.4)	113 (3.0)	<0.001
	ESRD or CKD	1780 (0.5)	1494 (2.2)	<0.001	64 (0.8)	132 (3.5)	<0.001
	COPD	6180 (1.7)	5999 (8.8)	<0.001	310 (3.7)	546 (14.4)	<0.001
	History of malignant neoplasm	21,462 (5.8)	8421 (12.4)	<0.001	747 (8.8)	576 (15.2)	<0.001
	Peptic ulcer disease history	153,885 (41.5)	37,049 (54.4)	<0.001	3768 (44.6)	2238 (59.1)	<0.001
Prescribed drugs		0.0 [0.0; 1.0]	7.0 [5.0; 9.0]	<0.001	0.0 [0.0; 2.0]	7.0 [ 6.0;10.0]	<0.001
Polypharmacy group				<0.001			<0.001
	Moderate polypharmacy (5 drugs)	0.0 (0.0)	42,132 (61.9)		0.0 (0.0)	1996 (52.7)	
	Severe polypharmacy (10 drugs)	0.0 (0.0)	25,926 (38.1)		0.0 (0.0)	1788 (47.3)	

Values are expressed as median (25th and 75th percentiles) or number (%). AF, atrial fibrillation; BP, blood pressure; CKD, chronic kidney disease; ESRD, end-stage renal disease; ICD, implantable cardioverter-defibrillator; MI, myocardial infarction; TIA, transient ischemic attack; COPD, chronic obstructive pulmonary disease; BMI, body mass index.

In terms of medication use, all prescribed drugs were significantly more commonly used by patients exhibiting polypharmacy. In the new-onset AF group, the most commonly prescribed drug was aspirin, subsequently by angiotensin-converting enzyme (ACE) inhibitors and angiotension II receptor blockers (ARBs). Diuretics were frequently prescribed to patients with polypharmacy, whereas lipid-lowering agents were more commonly prescribed to those without polypharmacy (Table 2).

**Table 2.** **Prescribed drugs between no polypharmacy and polypharmacy of No AF and new-onset AF**.

	No AF			New-onset AF		
	No polypharmacy	Polypharmacy	*p* value	No polypharmacy	Polypharmacy	*p* value
	(N = 371,069)	(N = 68,058)		(N = 8457)	(N = 3784)	
Aspirin	69,225 (18.7)	34,471 (50.6)	<0.001	4589 (54.3)	2753 (72.8)	<0.001
P2Y12 inhibitor	14,938 (4.0)	10,524 (15.5)	<0.001	1492 (17.6)	1201 (31.7)	<0.001
OAC	1055 (0.3)	525 (0.8)	<0.001	1690 (20.0)	804 (21.2)	0.114
Statin	106,965 (28.8)	36,613 (53.8)	<0.001	3689 (43.6)	2236 (59.1)	<0.001
Beta blocker	44,045 (11.9)	23,770 (34.9)	<0.001	3549 (42.0)	2295 (60.7)	<0.001
ACEI/ARB	84,383 (22.7)	36,480 (53.6)	<0.001	3855 (45.6)	2621 (69.3)	<0.001
DHP CCB	80,523 (21.7)	34,477 (50.7)	<0.001	2966 (35.1)	2224 (58.8)	<0.001
Non-DHP CCB	4548 (1.2)	3700 (5.4)	<0.001	821 (9.7)	587 (15.5)	<0.001
Diuretics	71,299 (19.2)	33,969 (49.9)	<0.001	3461 (40.9)	2561 (67.7)	<0.001
K Sparing diuretics	3845 (1.0)	3502 (5.1)	<0.001	799 (9.4)	632 (16.7)	<0.001
Alpha blocker	12,938 (3.5)	6910 (10.2)	<0.001	661 (7.8)	576 (15.2)	<0.001
Digoxin	866 (0.2)	1109 (1.6)	<0.001	1014 (12.0)	665 (17.6)	<0.001

Values are expressed as number (%). AF, atrial fibrillation; ACEI, angiotensin-converting enzyme inhibitor; ARB, angiotensin II receptor blocker; CCB, calcium Channel Blocker; DHP, dihydropyridine; OAC, oral anticoagulant.

### 3.2 Clinical Risk Factors for Polypharmacy in the New-Onset AF Group

In our investigation, age (per 10 years, OR 1.32; *p* < 0.001), heart failure (OR 1.95; *p* < 0.001), hypertension (OR 4.00; *p* <0.001), diabetes mellitus (OR 3.25; *p* < 0.001), ischemic stroke or transient ischemic attack (TIA) history (OR 2.36; *p* < 0.001), previous myocardial infarction (MI) (OR 1.54; *p* = 0.011), vascular disease (OR 1.29; *p* = 0.026), hyperthyroidism (OR 1.35; *p* = 0.011), osteoporosis (OR 1.67; *p* < 0.001), dyslipidemia (OR 1.80; *p* < 0.001), dementia history (OR 2.30; *p* = 0.035), peptic ulcer disease history (OR 1.25; *p* < 0.001), end-stage renal disease (ESRD) or chronic kidney disease (CKD) (OR 1.97; *p* < 0.001), chronic obstructive pulmonary disease (COPD) (OR 3.00; *p* < 0.001), and history of malignant neoplasm (OR 1.35; *p* < 0.001) were found to be independently associated with polypharmacy. These findings suggest that polypharmacy is primarily associated with multimorbidity and chronic diseases (Table 3).

**Table 3.** **Risk factors for polypharmacy in no AF and new-onset AF group**.

	No AF			New-onset AF		
	OR	95% CI	*p*-value	OR	95% CI	*p*-value
Age, per 10 years	1.45	1.44–1.47	<0.001	1.32	1.26–1.40	<0.001
Male	1.12	1.10–1.15	<0.001	1.11	1.00–1.24	0.054
Heart failure	1.73	1.66–1.81	<0.001	1.95	1.69–2.26	<0.001
Hypertension	4.00	3.92–4.09	<0.001	4.00	3.62–4.43	<0.001
Diabetes mellitus	3.81	3.71–3.92	<0.001	3.25	2.86–3.70	<0.001
Ischemic stroke/TIA	2.60	2.51–2.69	<0.001	2.36	2.03–2.73	<0.001
Previous MI	1.48	1.35–1.62	<0.001	1.54	1.11–2.15	0.011
Vascular disease	1.77	1.68–1.87	<0.001	1.29	1.03–1.62	0.026
Hyperthyroidism	1.29	1.22–1.36	<0.001	1.35	1.07–1.71	0.011
Hypothyroidism	1.46	1.38–1.53	<0.001			
Osteoporosis	1.87	1.82–1.92	<0.001	1.67	1.47–1.90	<0.001
Dyslipidemia	1.95	1.91–1.99	<0.001	1.80	1.63–1.98	<0.001
Dementia history	1.57	1.33–1.86	<0.001	2.30	1.06–4.98	0.035
Pacemaker or ICD implantation				0.09	0.01–0.78	0.029
Peptic ulcer disease history	1.24	1.21–1.26	<0.001	1.25	1.14–1.38	<0.001
Hemorrhagic stroke history	1.37	1.22–1.55	<0.001			
Major bleeding history	1.25	1.15–1.35	<0.001			
ESRD or CKD	1.51	1.39–1.65	<0.001	1.97	1.38–2.82	<0.001
COPD	3.06	2.93–3.20	<0.001	3.00	2.51–3.57	<0.001
History of malignant neoplasm	1.39	1.35–1.44	<0.001	1.35	1.17–1.56	<0.001
Economic status	0.97	0.97–0.98	<0.001	0.98	0.96–0.99	0.002

AF, atrial fibrillation; OR, odds ratio; CI, confidence interval; CKD, chronic kidney disease; COPD, chronic obstructive pulmonary disease; ESRD, end-stage renal disease; ICD, implantable cardioverter-defibrillator; MI, myocardial infarction; TIA, transient ischemic attack.

### 3.3 Polypharmacy and Adverse Clinical Outcomes

**Fig. 2.**
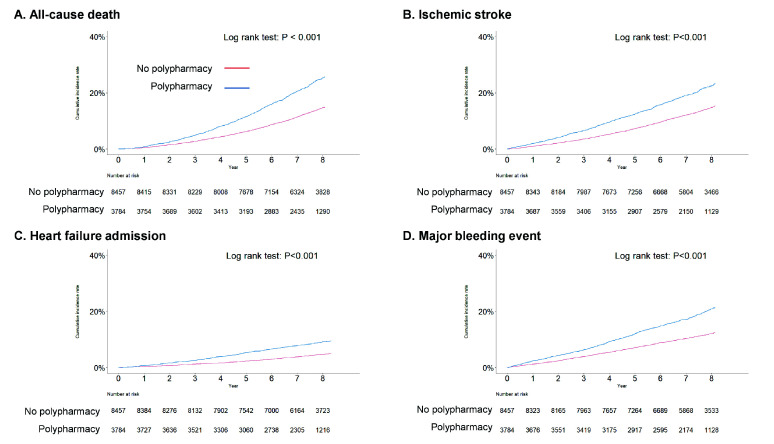
**Cumulative incidence rate curves for the clinical outcomes in the new-onset AF patients with or without polypharmacy**. Primary (A) and secondary outcomes (B–D) showed higher incidence in polypharmacy group in the new-onset AF population. AF, atrial fibrillation.

The incidence of adverse events was higher in the AF population than in the no AF population, both with and without polypharmacy. Significant difference between with and without polypharmacy were found in the no AF population (1.45 vs. 0.48 per 100 per years (PYs); *p* < 0.001) and patients with new-onset AF (6.21 vs. 3.91 per 100 PYs; *p* < 0.001). The incidence of all-cause mortality was higher in the new-onset AF population with polypharmacy by Kaplan-Meier survival analysis (log-rank *p* < 0.001) (Fig. 2). Similarly, through Kaplan-Meier analysis, it was shown that as the degree of polypharmacy increased, the rate of all-cause mortality was higher in the new-onset AF population (log-rank *p* < 0.001) (**Supplementary Fig. 1**). Nonetheless, multivariable Cox regression analyses showed that polypharmacy act as an independent risk factor for all-cause mortality (aHR 1.35, 95% CI 1.30–1.40; *p* < 0.001) in the population without AF, but not in the new-onset AF population.

Among the secondary outcomes in the new-onset AF population, the incidences of ischemic stroke/TIA (6.10 vs. 4.09 per 100 PYs; *p* < 0.001), major bleeding events (5.65 vs. 3.44 per 100 PYs; *p* < 0.001), and heart failure admission (2.37 vs. 1.27 per 100 PYs; *p* < 0.001) were significantly higher among patients with polypharmacy. Based on Kaplan-Meier survival analyses, the incidences of clinical events including major bleeding events, stroke, hospitalization due to heart failure were higher in the new-onset AF population with polypharmacy (log-rank *p* < 0.001 for all outcomes) (Fig. 2). Also, the degree of polypharmacy increased, the degree of major bleeding events, stroke, heart failure hospitalization were increased in the new-onset AF population (log-rank *p* < 0.001 for all outcomes) (**Supplementary Fig. 1**). Multivariable Cox regression analyses revealed that polypharmacy was associated with independently increased risk for ischemic stroke/TIA (aHR 1.27, 95% CI 1.21–1.33; *p* < 0.001), major bleeding (aHR 1.34, 95% CI 1.28–1.39; *p* < 0.001), and heart failure admission (aHR 1.58, 95% CI 1.37–1.81; *p* < 0.001) in the no AF population. However, Cox regression analysis indicated that polypharmacy was associated with independently increased risk for only major bleeding events (aHR 1.26, 95% CI 1.12–1.41; *p* < 0.001); thus, the risk of stroke and heart failure admission did not change in the new-onset AF population (Table 4).

**Table 4.** **Clinical outcomes in no-AF and new-onset AF population with or without polypharmacy with Time varying method**.

			No polypharmacy			Polypharmacy			Hazard ratio	*p* value	*p*-interaction
			No of events	Person years	Event rate	No of events	Person years	Event rate			
Primary outcome											
	All-cause death										<0.001
		No AF	13,323	27,585	0.48	7046	4873	1.45	1.35 (1.30–1.40)	<0.001	
		AF	1098	281	3.91	778	125	6.21	1.11 (0.99–1.24)	0.066	
Secondary outcome											
	Ischemic stroke										<0.001
		No AF	6720	26,918	0.25	3780	4557	0.83	1.27 (1.21–1.33)	<0.001	
		AF	1038	254	4.09	654	107	6.10	0.96 (0.86–1.09)	0.551	
	Major bleeding event										<0.001
		No AF	11,702	26,766	0.44	4806	4530	1.06	1.34 (1.28–1.39)	<0.001	
		AF	880	256	3.44	616	109	5.65	1.26 (1.12–1.41)	<0.001	
	Heart failure admission										<0.001
		No AF	561	27,105	0.02	557	4652	0.12	1.58 (1.37–1.81)	<0.001	
		AF	346	272	1.27	279	118	2.37	1.07 (0.88–1.29)	0.493	

AF, atrial fibrillation. Multivariable model adjusted for: Age, Male sex, Heart failure, Hypertension, Diabetes mellitus, Ischemic stroke or transient ischemic attack, Previous myocardial infarction, Dyslipidemia, Dementia, Hypothyroidism, Osteoporosis, Hemorrhagic stroke history, Major bleeding history, End-stage renal disease or chronic kidney disease, Chronic obstructive pulmonary disease, History of malignant neoplasm, Peptic ulcer disease.

### 3.4 Subgroup Analysis of AF Patients with High ${\mathrm{CHA}}_{2}$${\mathrm{DS}}_{2}$-VASc and HAS-BLED Scores, and Oral Anticoagulants (OACs) Status

In high risk patients, as defined by ${\mathrm{CHA}}_{2}$${\mathrm{DS}}_{2}$-VASc score 2 or higher and HAS-BLED score 3 or higher, the five important risk factors related to polypharmacy were hypertension, diabetes mellitus, ischemic stroke or TIA history, dementia history, and COPD (**Supplementary Table 3**). Polypharmacy in AF patients with higher thromboembolic risk with ${\mathrm{CHA}}_{2}$DS${\mathrm{DS}}_{2}$-VASc score 2 or higher showed increased risk for major bleeding events (aHR 1.20, 95% CI 1.05–1.37; *p* = 0.007) and heart failure admission independently (aHR 1.20, 95% CI 1.05–1.33; *p* = 0.007). In patients with OAC, polypharmacy independently contributed to increased risk of major bleeding events (aHR 1.51, 95% CI 1.21–1.88; *p* < 0.001) and heart failure admission (aHR 1.18, 95% CI 1.03–1.35; *p* = 0.013) (**Supplementary Table 4**).

## 4. Discussion

According to the findings of this retrospective ‘real-world’ study, polypharmacy is more common in the AF population, especially among those with comorbidities, than in the no AF population, and increases the incidence rate of adverse clinical outcomes. However, the risk of polypharmacy for adverse clinical outcomes was found to be lower in patients with AF, particularly those with high comorbidities represented by the ${\mathrm{CHA}}_{2}$${\mathrm{DS}}_{2}$-VASc and HAS-BLED scores, than in patients without such conditions, thereby opposing the findings of previous studies. This implies that appropriate medication use in patients with AF is as important as de-prescription for reducing polypharmacy. Polypharmacy, which can vary by definition, is generally known to occur in approximately 30% of patients older than 65 and approximately 52–64% of patients with AF [19, 20, 21].

### 4.1 High Adverse Events in Anticoagulated Patients with Polypharmacy

According to prior studies, patients on anticoagulation therapy with polypharmacy have an enhanced likelihood of experiencing adverse events, such as bleeding and mortality [7, 9, 22]. A Rivaroxaban versus Warfarin in Nonvalvular Atrial Fibrillation (ROCKET) trial sub study further demonstrated that major or non-major bleeding events are more common in patients with polypharmacy (aHR 1.47, 95% CI 1.31–1.65) [23]. Consistently, our results revealed a correlation between polypharmacy and major bleeding event, which could be relevant if antiplatelets contribute to the polypharmacy. Notably, the combination of antiplatelet therapy and OACs increases the risk of bleeding [24]. Additionally, we found a correlation between polypharmacy and increased thrombotic events, which suggests that polypharmacy is linked to an increased risk of both bleeding and thrombosis.

As vitamin K antagonists (VKAs) interact with several medications, achieving and maintaining a therapeutic international normalized ratio (INR) are challenging tasks, resulting in unpredictable dose effect and an increased likelihood of thromboembolic or bleeding complications [25, 26]. In the Relevance of Polypharmacy for Clinical Outcome in Patients Receiving Vitamin K Antagonists (ThrombEVAL study), individuals taking more than five drugs exhibited a reduced Time in Therapeutic Range (TTR), increased fluctuation in INR levels, and increased bleeding, hospitalization, and all-cause mortality risk compared to those without polypharmacy [9]. Similarly, we found that patients with polypharmacy had a lower TTR and showed a elevated risk of poor clinical outcomes. Non-vitamin K antagonist oral anticoagulants (NOACs) might reduce the compound risk of drug interactions during anticoagulation therapy, due to their lower tendency for drug-drug interactions [27]. Indeed, analysis of combined data from Medicaid, US commercial claims, and Medicare showed that among AF patients receiving polypharmacy, those prescribed NOACs exhibited a lower incidence and risk of adverse events compared to those on VKAs [28]. The association between polypharmacy and adverse effects exhibits a sophisticated and multifactorial mechanism. Patients with AF often present various cardiovascular risk factors and comorbidities, leading to the prescription of numerous medications. Consequently, polypharmacy can be considered as an indicator of multimorbidity, and our findings clearly demonstrated its independent association with comorbidities, including hypertension, diabetes vascular disease, and heart failure.

### 4.2 Management of Patients with Polypharmacy

Polypharmacy could be an indicator of health conditions in patients with AF, distinguishing patients with a high-risk profile due to multiple coexisting medical conditions and assisting in the identification of frail patients. In addition to preventing stroke, it is crucial to manage symptoms and cardiovascular risk factors in patients with AF. In fact, the presence of multiple concomitant conditions is common in AF patients, leading to poorer quality of life and clinical outcomes [29]. The ABC pathway, an all-inclusive and coordinated strategy for AF management that involves stroke prevention with anticoagulation therapy (A), symptom control which includes rate control and rhythm management (B), and management of cardiovascular comorbidities and concomitant risk factors (C), could be exploited to aptly manage AF [30].

Patients with polypharmacy require specialized and intensive monitoring that focuses on their special requirements. It is crucial to carefully examine prescriptions to detect potential pharmacological interactions, assess the risks and benefits of each medication, and implement detailed monitoring plans. Implementing strategies to decrease unnecessary prescriptions or discontinue unnecessary lifelong medication is also important. Decreasing the number of prescribed medications could lead to a reduction in adverse events [21, 31]. For example, implementing specific approaches to cease the use of unneeded antiplatelet agents can help lowering the risk of bleeding [32]. Furthermore, the adoption of polypills might simplify therapeutic adherence and reduce the daily pill burden [33].

### 4.3 Limitations

Our study had several limitations. As this study had a retrospective nature and was performed using Korean NHIS-HealS cohort, Korean patients added to the cohort between 2002 and 2009 were analyzed in this study. Consequently, the use of antiplatelet agents as a stroke prevention option, the relatively low ${\mathrm{CHA}}_{2}$DS${\mathrm{DS}}_{2}$-VASc scores in the population, and Warfarin as the only available OAC option at that time, known to increase the risk of bleeding events in Asian populations, contributed to the relatively low OAC prescription rate of around 20% in the new-onset AF group. The use of VKAs was relatively high and the proportion of NOAC usage was low. These factors may influence the effect of polypharmacy on the occurrence of major bleeding events.

## 5. Conclusions

By employing a ‘real-world’ AF cohort, we presented the clinical impact of polypharmacy on patients with AF. Although the rate of adverse outcomes is elevated in the polypharmacy group, contrary to previous studies, polypharmacy is not independently associated with poor outcomes, except in instances of major bleeding event. Such findings might be due to the concomitant increase in comorbidities, which also elevates the risk in the population without polypharmacy. In patients with AF, not only the efforts to reduce polypharmacy, but also the application of appropriately tailored medications for concurrent conditions, such as comorbidities, should be considered.

## Data Availability

The data sets generated and/or analyzed during the current study are not publicly available due to privacy concerns the policies of the Korea National Health Insurance Service Data but are available from the Korea National Health Insurance Service on reasonable request.

## Supplementary Material

Supplementary material associated with this article can be found, in the online version, at https://doi.org/10.31083/j.rcm2505164.
