# Supplementary material for: Clinical Implications of Polypharmacy for Patients with New-Onset Atrial Fibrillation Based on Real-World Data: Observations from the Korea National Health Insurance Service Data
Source: Rev Cardiovasc Med. 2024 May 11;25(5):164. doi: 10.31083/j.rcm2505164 (PMC11267179; doi:10.31083/j.rcm2505164)
Supplement: Supplementary file 1 [file 2153-8174-25-5-164-s1.docx]

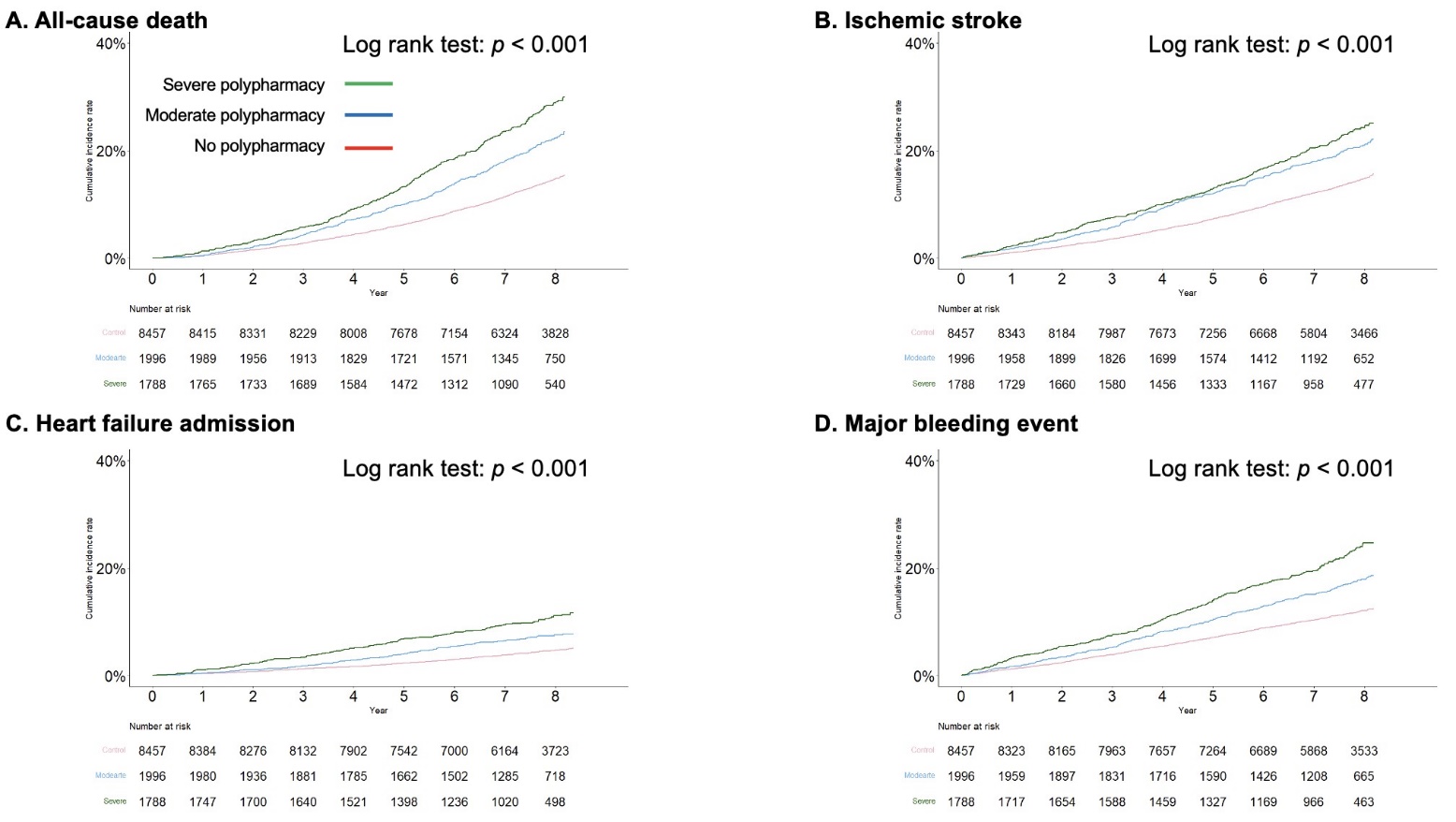


**Supplementary Fig. 1. Cumulative incidence rate curves for the clinical outcomes in patients with severe polypharmacy, moderate polypharmacy, or without polypharmacy.**

Supplementary Table 1. Definitions and ICD-10 codes employed to define medical conditions, associated comorbidities, and the medications and procedures for the treatment of atrial fibrillation..

|  | **Definitions** | **Codes or conditions** |
| --- | --- | --- |
| **Medical conditions** | |  |
| Atrial fibrillation | Defined from diagnosis* | I48 |
| Heart failure | Defined from diagnosis* | ICD-10: I11.0, I50, I97.1 |
| Heart failure admission history | Defined from principal or first secondary admission diagnoses of heart failure | ICD-10: I11.0, I50, I97.1 |
| Hypertension | Defined if fulfilling both diagnosis* and treatment within 90 days prior to the first recorded prescription or procedure for rhythm or rate control | ICD-10: I10, I11, I12, I13, I15  Treatment: prescription for at least one of all kinds of antihypertensive medication |
| Diabetes mellitus | Defined if fulfilling both diagnosis* and treatment within 90 days prior to the first recorded prescription or procedure for rhythm or rate control | ICD-10: E10, E11, E12, E13, E14  Treatment: prescription for at least one of all kinds of oral antidiabetics or insulin |
| Dyslipidemia | Defined from diagnosis* | ICD-10: E78 |
| Ischemic stroke | Defined from diagnosis* | ICD-10: I63, I64 |
| Transient ischemic attack | Defined from diagnosis* | ICD-10: G45 |
| Intracranial bleeding | Defined from diagnosis* | ICD-10: I60, I61, I62 |
| Myocardial infarction | Defined from diagnosis* | ICD-10: I21, I22, I25.2 |
| Peripheral arterial disease | Defined from diagnosis* | ICD-10: I70.0, I70.1, I70.2, I70.8, I70.9 |
| Valvular heart disease | Defined from diagnoses* mitral stenosis or claims for heart valve surgery | ICD-10: I05.0, I05.2, I34.2, Z95.2-4  Claim for valve replacement or valvuloplasty: O1781, O1782, O1783, O1791, O1792, O1793, O1797, O1794, O1795, O1796, O1798 |
| Chronic kidney disease | Defined from eGFR or diagnosis*  (if laboratory value was not available, diagnosis code was used) | eGFR <60mL/min per 1.73 m^2^  ICD-10: N18, N19 |
| Hyperthyroidism | Defined from diagnosis* | ICD-10: E05 |
| Hypothyroidism | Defined from diagnosis* | ICD-10: E03 |
| Malignancy | Defined from diagnoses* of cancer (non-benign) | ICD-10: C00-C97 |
| Chronic obstructive pulmonary disease | Defined if fulfilling both diagnosis* and treatment within 90 days prior to the first recorded prescription or procedure for rhythm or rate control | ICD-10: J42, J43(except J43.0), J44  Treatment: SABA, SAMA, LABA, LAMA, ICS, ICS+LABA, or methylxanthine (>1 months). |
| Osteoporosis | Defined from diagnosis* | ICD-10: M80, M81, M82 (except M82.0) |
| **Drug treatment for atrial fibrillation (available in South Korea)** | | |
| Anti-arrhythmic drug | |  |
| Class Ic |  | flecainide, pilsicainide, propafenone |
| Class III |  | amiodarone, dronedarone, sotalol |
| Beta-blocker |  | atenolol, bisoprolol, carvedilol,metoprolol, nebivolol, propranolol, labetalol |
| Calcium channel blocker | | diltiazem, verapamil |
| Cardiac glycosides |  | digoxin |

*For greater accuracy, either one diagnosis during hospitalization or more than twice at outpatient clinics was requited for the diagnosis.

Supplementary Table 2. Definitions and codes used for study outcomes.

| **Outcomes** | **Definitions** | **Codes or conditions** | **PPV** |
| --- | --- | --- | --- |
| Ischemic stroke | Defined from admission diagnosis with concomitant imaging studies of the brain or related death | ICD-10: I63, I64 | 90.6%*  (2347/2591) |
| Hospitalization owing to heart failure | Defined from principal or first secondary admission diagnoses of heart failure | ICD-10: I11.0, I50, I97.1 | 82.1%*  (110/134) |
| Intracranial bleeding | Defined from admission diagnosis with concomitant imaging studies of the brain or related death | ICD-10: I60-I62 | 87.5%*  (286/327) |
| Gastrointestinal bleeding | Defined from admission diagnosis or related death | ICD-10: K25-28 (subcodes 0-2 and 4-6 only), K62.5, K92.0, K92.1, K92.2, I85.0, I98.3 | 92.0%‡  (184/200) |

PPV was represented as % (number of true positive cases / number of examined cases).
*Validated in a study by Kim, D. et al. (Treatment timing and the effects of rhythm control strategy in patients with atrial fibrillation: nationwide cohort study. *BMJ* 2021;373:n991).

ICD-10, International Classification of Diseases-10th Revision; PPV, positive predictive value.

Supplementary Table 3. Clinical risk factors for polypharmacy in patients with new-onset AF with CHA_2_DS_2_-VASc score 2 or higher and HAS-BLED score 3 or higher.

|  | **CHA_2_DS_2_-VASc** ≥ **2** | | | **HAS-BLED** ≥**3** | | |
| --- | --- | --- | --- | --- | --- | --- |
|  | **OR** | **95% CI** | ***p*-value** | **OR** | **95% CI** | ***p* -value** |
| Age, per 10years | 1.23 | 1.14 – 1.32 | <0.001 |  |  |  |
| Male | 1.19 | 1.04 – 1.35 | 0.009 | 1.20 | 0.99 – 1.46 | 0.068 |
| Heart failure | 1.93 | 1.66 – 2.24 | <0.001 | 2.07 | 1.66 – 2.56 | <0.001 |
| Hypertension | 3.89 | 3.41 – 4.43 | <0.001 | 2.40 | 1.49 – 3.85 | <0.001 |
| Diabetes mellitus | 3.15 | 2.73 – 3.63 | <0.001 | 2.97 | 2.37 – 3.74 | <0.001 |
| Ischemic stroke/TIA | 2.27 | 1.96 – 2.64 | <0.001 | 1.85 | 1.52 – 2.25 | <0.001 |
| Previous MI | 1.54 | 1.10 – 2.14 | 0.011 |  |  |  |
| Vascular disease | 1.23 | 0.98 – 1.54 | 0.076 | 1.47 | 1.14 – 1.90 | 0.003 |
| Hyperthyroidism |  |  |  | 1.33 | 0.90 – 1.98 | 0.157 |
| Osteoporosis | 1.65 | 1.44 – 1.90 | <0.001 | 1.82 | 1.46 – 2.26 | <0.001 |
| Dyslipidemia | 1.78 | 1.58 – 2.00 | <0.001 | 1.85 | 1.56 – 2.20 | <0.001 |
| Dementia history | 2.39 | 1.11 – 5.15 | 0.026 | 2.17 | 0.84 – 5.64 | 0.111 |
| Pacemaker or ICD implantation | 0.11 | 0.01 – 1.01 | 0.051 | 0.15 | 0.01 – 1.63 | 0.119 |
| Peptic ulcer disease history | 1.31 | 1.17 – 1.47 | <0.001 | 1.33 | 1.12 – 1.58 | 0.001 |
| ESRD or CKD | 1.89 | 1.25 – 2.84 | 0.002 | 1.68 | 1.11 – 2.56 | 0.015 |
| COPD | 2.81 | 2.31 – 3.43 | <0.001 | 2.55 | 1.88 – 3.46 | <0.001 |
| History of malignant neoplasm | 1.22 | 1.03 – 1.44 | 0.021 |  |  |  |
| Economic status | 0.99 | 0.97 – 1.00 | 0.138 |  |  |  |

CKD, Chronic kidney disease; COPD, Chronic obstructive pulmonary disease; ESRD, End-stage renal disease; ICD, Implantable cardioverter-defibrillator; MI, Myocardial infarction; TIA, Transient ischemic attack

Supplementary Table 4. Clinical outcomes in AF patients with or without polypharmacy according to difference CHA_2_DS_2_-VASc, HAS-BLED score, and OAC status.

|  | **No polypharmacy** | | | **Polypharmacy** | | | **Hazard ratio** | ***p*-value** | ***p*-interaction** |
| --- | --- | --- | --- | --- | --- | --- | --- | --- | --- |
|  | **No of events** | **Person years** | **Event  rate** | **No of events** | **Person years** | **Event  rate** |  |  |  |
| ***CHA*_2_*DS*_2_*-VASc score*** |  |  |  |  |  |  |  |  |  |
| All-cause mortality |  |  |  |  |  |  |  |  | 0.201 |
| ≤1 | 423 | 171 | 2.47 | 87 | 24 | 3.66 | 1.37 (1.07 – 1.74) | 0.010 |  |
| ≥2 | 675 | 110 | 6.15 | 691 | 102 | 6.80 | 1.05 (0.93 – 1.19) | 0.410 |  |
| Ischemic stroke |  |  |  |  |  |  |  |  | 0.254 |
| ≤1 | 431 | 159 | 2.71 | 75 | 22 | 3.46 | 1.12 (0.86 – 1.45) | 0.398 |  |
| ≥2 | 607 | 95 | 6.39 | 579 | 86 | 6.76 | 0.93 (0.82 – 1.06) | 0.292 |  |
| Major bleeding event |  |  |  |  |  |  |  |  | 0.839 |
| ≤1 | 417 | 158 | 2.64 | 87 | 21 | 4.12 | 1.49 (1.16 – 1.90) | 0.001 |  |
| ≥2 | 463 | 98 | 4.73 | 529 | 88 | 6.01 | 1.20 (1.05 – 1.37) | 0.007 |  |
| Heart failure admission |  |  |  |  |  |  |  |  | 0.874 |
| ≤1 | 108 | 168 | 0.64 | 19 | 23 | 0.82 | 1.33 (0.79 – 2.23) | 0.280 |  |
| ≥2 | 238 | 104 | 2.29 | 260 | 95 | 2.75 | 1.20 (1.05 – 1.33) | 0.007 |  |
| ***HAS-BLED score*** |  |  |  |  |  |  |  |  |  |
| All-cause mortality |  |  |  |  |  |  |  |  | 0.411 |
| ≤2 | 868 | 249 | 3.49 | 348 | 69 | 5.04 | 1.13 (0.98 – 1.29) | 0.081 |  |
| ≥3 | 230 | 32 | 7.15 | 430 | 56 | 7.64 | 1.08 (0.91 – 1.29) | 0.363 |  |
| Ischemic stroke |  |  |  |  |  |  |  |  | 0.304 |
| ≤2 | 784 | 228 | 3.44 | 224 | 64 | 3.52 | 0.87 (0.74 – 1.01) | 0.070 |  |
| ≥3 | 254 | 26 | 9.72 | 430 | 44 | 9.84 | 1.00 (0.85 – 1.19) | 0.980 |  |
| Major bleeding event |  |  |  |  |  |  |  |  | 0.041 |
| ≤2 | 714 | 228 | 3.13 | 303 | 61 | 4.99 | 1.41 (1.22 – 1.63) | <0.001 |  |
| ≥3 | 166 | 28 | 5.95 | 313 | 48 | 6.47 | 0.98 (0.80 – 1.20) | 0.836 |  |
| Heart failure admission |  |  |  |  |  |  |  |  | 0.670 |
| ≤2 | 246 | 243 | 1.01 | 97 | 66 | 1.46 | 1.12 (0.85 – 1.47) | 0.418 |  |
| ≥3 | 100 | 30 | 3.36 | 182 | 51 | 3.53 | 1.01 (0.77 – 1.32) | 0.945 |  |
| ***OAC status*** |  |  |  |  |  |  |  |  |  |
| All-cause mortality |  |  |  |  |  |  |  |  | 0.623 |
| No OAC | 878 | 224 | 3.92 | 630 | 98 | 6.40 | 1.09 (0.97 – 1.24) | 0.150 |  |
| OAC | 220 | 57 | 3.85 | 148 | 27 | 5.49 | 1.11 (0.86 – 1.43) | 0.427 |  |
| Ischemic stroke |  |  |  |  |  |  |  |  | 0.006 |
| No OAC | 540 | 211 | 2.56 | 396 | 88 | 4.50 | 1.03 (0.88 – 1.21) | 0.700 |  |
| OAC | 498 | 43 | 11.53 | 258 | 19 | 13.37 | 0.95 (0.80 – 1.14) | 0.607 |  |
| Major bleeding event |  |  |  |  |  |  |  |  | 0.193 |
| No OAC | 686 | 204 | 3.37 | 467 | 86 | 5.45 | 1.18 (1.03 – 1.35) | 0.014 |  |
| OAC | 194 | 52 | 3.73 | 149 | 23 | 6.39 | 1.51 (1.21 – 1.88) | <0.001 |  |
| Heart failure admission |  |  |  |  |  |  |  |  | 0.267 |
| No OAC | 205 | 219 | 0.94 | 182 | 93 | 1.95 | 1.10 (0.86 – 1.40) | 0.450 |  |
| OAC | 141 | 53 | 2.65 | 97 | 25 | 3.95 | 1.18 (1.03 – 1.35) | 0.013 |  |

AF, Atrial fibrillation; CI, Confidential interval
